# Genetic Dynamics and Dispersion Pattern Changes in *Aedes aegypti* Populations (Diptera: Culicidae) in Yunnan Province, China, over Three Consecutive Years

**DOI:** 10.3390/insects17101087

**Published:** 2026-10-20

**Authors:** Jian Gao, Heng-Duan Zhang, Zhi-Ming Wu, Qing-Ming Shi, Dan Xing, Xiao-Xia Guo, Chun-Xiao Li, Yan-De Dong, Tong-Yan Zhao, Hong-Liang Chu

**Affiliations:** 1 Department of Disinfection and Vector Control, Jiangsu Provincial Center for Disease Control and Prevention, Nanjing 210009, China; camgao_006@163.com (J.G.); wuzhiming2014@126.com (Z.-M.W.); 2 State Key Laboratory of Pathogen and Biosecurity, Beijing Institute of Microbiology and Epidemiology, Beijing 100071, China; zhanghengduan1987@163.com (H.-D.Z.);; 3 Center for Disease Control and Prevention in Western Theater Command, Lanzhou 730030, China

**Keywords:** mosquitoes, *Aedes aegypti*, genetic structure, invasion, *cox I*, microsatellite

## Abstract

*Aedes aegypti* is the primary vector of mosquito-borne viruses and threatens human health globally. To monitor temporal changes in the population genetics of *Ae. aegypti* in Yunnan Province, we collected specimens from five sites between 2016 and 2018 and analyzed them at nine microsatellite loci and the mitochondrial *cox I* gene. Genetic diversity in these populations rose initially and then declined over the study period. The inbreeding coefficient shifted from a heterozygous state toward moderate inbreeding. Ten *cox I* haplotypes were identified, with haplotypes H06 and H09 prevalent across regions, while other haplotypes were limited to specific regions or years. Clustering analyses demonstrated significant population shifts of *Ae. aegypti* across the five sampling sites from 2016 to 2018. AMOVA analysis of all *Ae. aegypti* populations indicated that from 2016 to 2018, the main source of variation was predominantly among individuals, accounting for 76.43%, 65.15%, and 80.36%, respectively. Population dispersion patterns across locations initially increased and then decreased over time. Therefore, continuous monitoring is essential, and particular attention should be paid to intra-provincial spread, especially between port and urban areas.

## Simple Summary

The yellow fever mosquito (*Aedes aegypti*), the main carrier of dengue fever and other mosquito-borne diseases, threatens human health worldwide. In China, this mosquito is currently restricted to Hainan Island, the Leizhou Peninsula, and Yunnan Province, which borders countries with frequent dengue outbreaks and is at high risk. To help prevent and control these diseases, we tracked the genetic dynamics and dispersion pattern changes in this mosquito at five sites across Yunnan Province from 2016 to 2018, using nine microsatellite markers and the *cox I* gene. We found that genetic diversity first rose and then declined. Two genetic variants dominated in nearly all areas and years, while others appeared only in certain places or years, and the genetic composition of local populations changed noticeably between years. Mosquitoes spread most widely in 2017, whereas their spread was reduced in 2018. Our findings show that continuous monitoring is essential, with special attention to mosquito movement within the province, especially between ports and cities. This knowledge can help health authorities focus surveillance and control efforts and protect public health.

## 1. Introduction

Mosquito-borne viruses, particularly dengue virus (DENV), Zika virus (ZIKV), and Chikungunya virus (CHIKV), present a significant global public health threat [1]. DENV alone impacts a proportion of the world’s population and infects millions annually [2]. Currently, no effective preventive vaccines exist for these diseases [3,4]. Therefore, ongoing surveillance and targeted control measures are crucial to safeguarding human health, particularly in tropical and subtropical areas where mosquito breeding is constant.

*Aedes aegypti* (Linnaeus, 1762), an invasive mosquito species of African origin, was dispersed globally through maritime trade from the 15th to the 17th century and has since become established across tropical and subtropical regions worldwide [5,6]. In China, permanent populations are currently restricted to Hainan Island, the Leizhou Peninsula, and the border regions of southern Yunnan, where human-mediated transport continues to facilitate local range expansion and sustain the risk of arbovirus transmission [7,8]. Unlike *Ae. albopictus* (Skuse, 1894), *Ae. aegypti* is predominantly endophilic, thrives in water-filled environments, and prefers to bite during the day [9,10]. Even at low densities, *Ae. aegypti* can trigger outbreaks of related arboviruses, particularly since they breed near human dwellings [4]. As an invasive species, *Ae. aegypti* demonstrates significant ecological niche plasticity, aiding its adaption to new environments and potentially allowing it to replace native mosquito species, thereby posing a heightened threat to public health [11,12]. This species is closely linked to urban settings, with its distribution influenced by climate and socioeconomic factors [13,14]. Due to urbanization in China and the impacts of global climate change, *Ae. aegypti*’s range is expanding northward from the south [15]. Consequently, real-time monitoring of its invasion and spread in China is crucial for the prevention and control of related arboviruses.

Yunnan province, located in southwestern China, shares borders with several Southeast Asian countries affected by DENV, ZIKV, and CHIKV, including Laos, Vietnam, and Myanmar. The ongoing tourism, trade, and labor exchanges between Yunnan and these nations have resulted in a consistent rise in imported arboviral infection cases [16,17]. Additionally, Yunnan’s geographical and climatic conditions are conducive to the breeding of *Ae. aegypti*, potentially exacerbating the severity of dengue fever outbreaks both locally and across the country. Consequently, monitoring the population dynamics of *Ae. aegypti* in this region is essential for effectively preventing and controlling dengue fever epidemics.

Assessing mosquito biodiversity, including genetic structuring and population dispersion, is vital for developing and implementing effective strategies to control mosquito populations, and consequently, mosquito-borne infectious diseases [18]. Among molecular markers, microsatellite loci and the mitochondrial gene *Cox I* are commonly used due to their codominance, polymorphism, and moderate evolutionary rate [19]. For example, Ambrose et al. [20] employed these markers to evaluate the population genetic structure of *Anopheles farauti*, discovering significant gene flow barriers among *An. farauti* populations in the Solomon Archipelago. Similarly, Li et al. (2020) utilized these markers to detect the genetic differentiation among the *Aedes aegypti* populations of Hainan and Leizhou Peninsula, China [21]. Yamashita et al. (2024) also applied these markers to confirm that Mozambican invasive mosquitoes originated from Southeast Asian sources [22].

Numerous studies have examined the population structure of *Ae. aegypti* [23,24,25,26,27], but few have analyzed the dynamics and dispersal patterns of the mosquito populations over consecutive years. This study monitored the *Ae. aegypti* populations in various regions of Yunnan Province during 2017 and 2018. By incorporating population data from 2016 [23], the research elucidated the population dynamics of *Ae. aegypti* in Yunnan Province over three years using nine microsatellite loci and the mitochondrial *cox I* gene. The findings aim to provide valuable insights for future dengue control efforts.

## 2. Materials and Methods

### 2.1. Mosquito Sampling and Processing

Given that this study was carried out within the framework of a single, continuously funded project in collaboration with Shi et al. (2017) [23], we extracted partial mitochondrial gene *cox I* and microsatellite data originally reported previously for inclusion in the present research. The principles governing the data extraction for *Ae. aegypti* in 2016 are outlined as follows: The Jinghong city sample comprised 120 specimens, with 20 samples collected from each of the six sampling sites in Jinghong in 2016 (NKH, YSC, DMY, LJY, PSS, and GSZ). A total of 50 samples were selected from the DLKA 2016 to constitute the Daluo port sample. In Mengla city, 29 and 30 samples were, respectively, chosen from ML-1 and ML-2 for the Mengla sample; for the Lincang area, 30 samples were taken from each of GM-1 and GM-2 to compile the Lincang sample. Lastly, Ruili city consisted of 150 specimens, with 20–30 samples gathered from each of the six sampling sites in Ruili in 2016 (MNL, JDL, HDH, JCG, GMG, and JGH) for subsequent analysis. *Ae. aegypti* mosquitoes were collected from three regions (Banna prefecture, Dehong prefecture, and Lincang area) across Yunnan Province from 2017 to 2018. Banna prefecture comprises three sampling sites: Jinghong city, Mengla city, and Daluo port. Jinghong city is situated at the core of Banna prefecture, abutting the Mekong River, whereas Mengla city and Daluo port are located along the border. Dehong prefecture contains one sampling site, Ruili city, which lies within a border region dedicated to agricultural and jewelry trade activities. The sampling site in the Lincang area is Lincang city, which is surrounded by mountains and remains relatively secluded from the outside world. Sustained sampling of *Ae. aegypti* specimens was carried out within a 400 m radius centered on the coordinates of each sampling site. A detailed description of the sampling biotopes, including location, main vegetation, soil type and water bodies, was summarized in Supplemental Material: Table S1. The central coordinates for each site, along with the annual quantity of mosquitoes collected, are presented in Table 1. Within a radius of roughly 400 m around the central coordinates of each site, we randomly select 1 to 6 small sampling points to collect *Ae. aegypti* larvae. In principle, each of these sampling points should yield no fewer than 30 larvae. Additionally, we strictly adhere to the sampling points chosen by Shi et al. (2017) [23] in 2017 and 2018 for our data analysis, ensuring the continuity of our sampling efforts (Supplemental Material: Figure S1). All larvae were transferred to the lab in isolated containers and maintained at 28 °C and 75% relative humidity with a constant photoperiod (14 h light/10 h dark) until emergence. The adults were then morphologically identified under a dissecting microscope using the taxonomic keys of Lu and Norbert [28,29]; the identified female *Ae. aegypti* were then stored in 95% ethanol at −80 °C until usage.

### 2.2. DNA Isolation, cox I Gene Amplification, and Microsatellite Genotyping

Based on the sample size data of each site, we randomly selected 50 to 60 adult mosquitoes from each site for subsequent molecular investigations. For sites with fewer than 60 mosquitoes, we processed the entire available mosquito population. For sites with more than 60 mosquitoes, we randomly selected 60 per site for further processing. According to the manufacturer’s protocol, all samples were milled with a freeze grinder, and nucleic acid was extracted using a fully automated nucleic acid extractor with the DNeasy Blood & Tissue Kit (Qiagen, Germantown, Maryland, USA). All DNA samples were stored at −80 °C.

Referring to Gao et al. [30], a 550 bp fragment of the *cox I* gene was amplified with the primers: 5′-GGAGGATTTGGAAATTGATTAGTTC-3′ (*cox I*-Forward) and 5′-CCCGGTAAAATTAAATATAAACTTC-3′ (*cox I*-Reverse). In total, 3.0 μL of mosquito DNA was amplified with a TAdvanced Thermal Cycler (Analytikjena, Jena, Germany) in a 50 μL reaction system, containing 5 μL PCR buffer (TaKaRa, Kusatsu, Japan), 4 μL dNTPs (2.5 mM each, TaKaRa, Japan), 1 μL primers (10 pmol/μL, TaKaRa, Japan), 0.5 μL PrimeSTAR HS DNA polymerase (TaKaRa, Japan), and 15.5 μL ddH20. The application program was set as pre-denaturation at 95 °C for 3 min, followed by 35 cycles of denaturation at 95 °C for 45 s, annealing at 55 °C for 30 s, and elongation at 72 °C for 1 min, with a final elongation at 72 °C for 7 min. All PCR products were detected by 1.2% agarose gel electrophoresis and sequenced on an ABI 3730XL automatic sequencer (Applied Biosystems, Foster City, CA, USA).

Referring to Shi et al. [23], nine microsatellite loci were selected for microsatellite genotyping. The forward primer sequence for each locus was end-labeled with one of the fluorescent dyes FAM, HEX, or TAMRA (Supplementary Material: Table S1). Individuals were genotyped based on the variation at these nine microsatellite loci using a TAdvanced Thermal Cycler (AnalytikJena, Germany), and the products were analyzed using an ABI 3730XL automatic sequencer (Applied Biosystems, USA). The data were analyzed using the software GeneMarker (Version 2.2.0). All data, along with those from Shi et al., were compiled into an Excel datasheet for the subsequent genetic analysis.

### 2.3. Genetic Diversity, Differentiation, and Population Clustering

Genetic diversity indices, such as the effective number of alleles (*ne*), observed heterozygosity (*Ho*), expected heterozygosity (*He*), and positive inbreeding coefficient (*F_IS_*), were calculated with the software popgen32 v.1.32. Based on the mitochondrial *cox I* gene, the haplotype was calculated using the software DnaSP v.6.12.03 [31]. Structure v.2.3.4 and Evanno’s *ΔK* method were employed to calculate the optimal number of populations (K) of each year. We set 200,000 burn-in iterations to achieve chain convergence, followed by 2,000,000 post-burn-in MCMC repetitions for parameter sampling. Ten independent replicate runs were conducted for each K. The optimal K value was then depicted with Distribute v. 1.1 [32]. A Discriminant Analysis of Principal Components (DAPC) was performed on the microsatellite dataset using the DAPC function in the R software package “Adegenet v.2.1.3” [33], the UPGMA tree was constructed with the software NTSYS pc v.2.2, and the polygenetic tree was modified with the software TreeView v.2.0 [34].

### 2.4. AMOVA Test, Haplotype Evolution and Distribution Analysis

Based on microsatellite data, genetic diversity and molecular variance, all *Ae. aegypti* populations were assessed using the Analyses of Molecular Variance (AMOVA) function in Arlequin v.3.5.2.2 [35]. Analyses of the genetic evolution and regional distribution of each haplotype were performed using BEAST v.1.8.4 [36] with the number of bootstrap replications set as 10,000, and the best BEAST model (K2P + G) was determined using JModelTest v.2.1.10 [37]. The TCS network was constructed using Network v.10.0.0.0 [38,39].

Based on the gene flow values across distinct *Ae. aegypti* populations, the dispersion routine across 5 sampling sites was evaluated using the divMigrate function in the R package “diversity v.1.9.90” [40], with guided replication values of 3 and 5 and an alpha value of 0.05. Meanwhile, Nei’s genetic identity (*I*) was used to display population similarity using the R package “Heatmap v.1.1.9” [41].

## 3. Results

### 3.1. Genetic Diversity Variation in Ae. aegypti Populations During 2016 to 2018

The microsatellite datasets indicate that the *ne* value for the *Ae. aegypti* population at each sample site ranged from 2.16 to 3.33 between 2016 and 2018. In 2016, population heterozygosity analysis results showed that the observed heterozygosity (*Ho*) for the *Ae. aegypti* population in Lincang city, Daluo port, and Mengla city (*Ho*, 0.61~0.65) surpassed the expected heterozygosity (*He*, 0.58~0.61). However, in 2017 and 2018, the Ho values for each *Ae. aegypti* population were lower than expected. Additionally, our analysis found that the inbreeding coefficient values for *Ae. aegypti* populations in Mengla city, Daluo port, and Lincang city in 2016 were negative, ranging from −0.07 to −0.04, while other populations showed positive inbreeding coefficients (Table 2). Furthermore, the *F_IS_* values for each population exhibited a trend of initially increasing and then decreasing over the years.

### 3.2. Haplotype Diversity and Composition Dynamics of All Ae. aegypti Populations Among Different Regions

**Figure 1:**
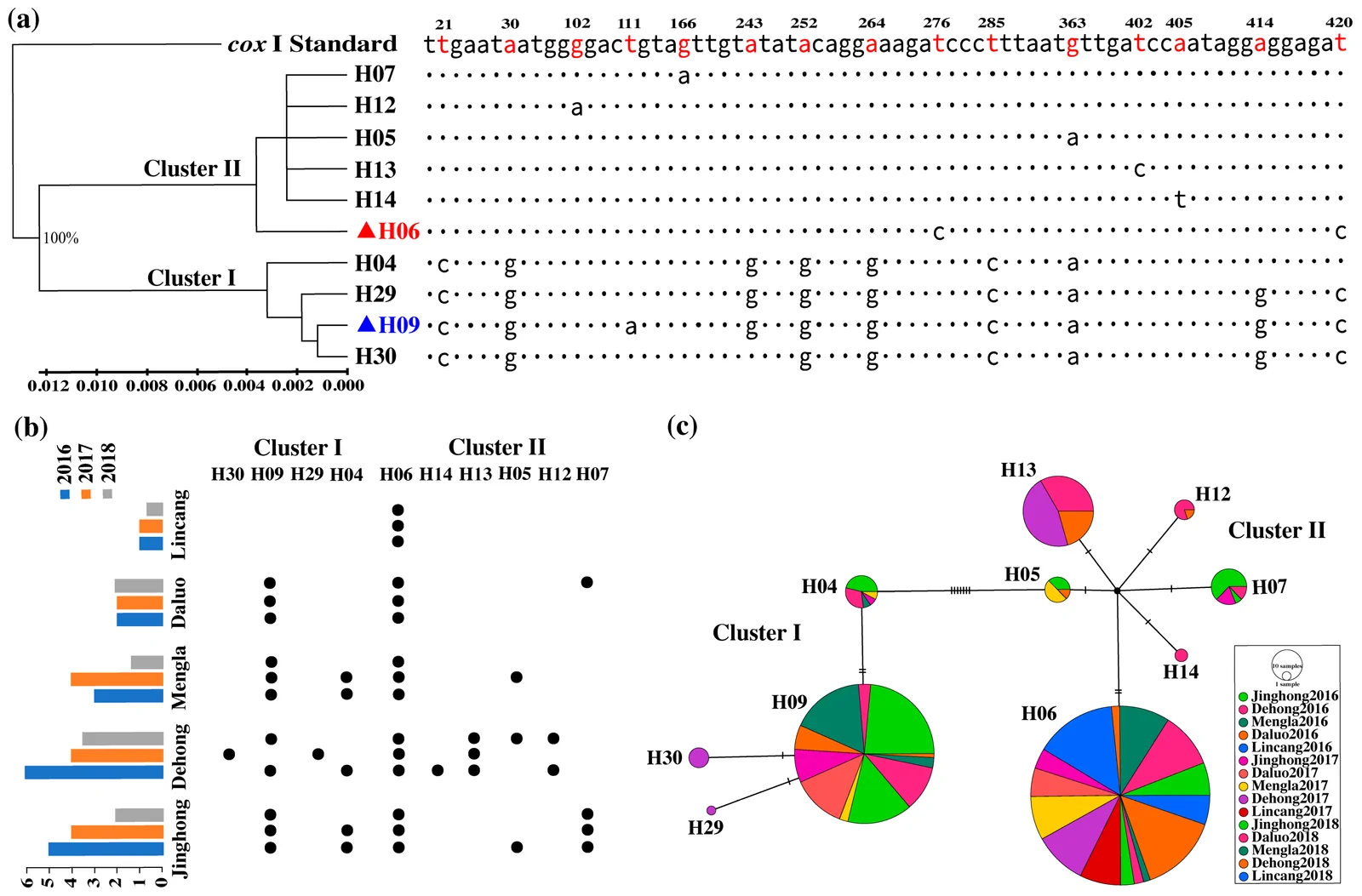
Haplotype diversity and composition dynamics of *Ae. aegypti* populations based on mitochondrial *cox I* gene. (**a**) The evolution tree of all ten predominant haplotypes, along with a display of their respective mutation sites in comparison to the standard sequence; (**b**) distribution variations in each haplotype across different sampling sites in various years; (**c**) TCS network of all haplotypes, displaying the evolutionary relationships among different haplotypes, along with their proportional distribution at each sample site across different years.

Using the mitochondrial *cox I* gene, a total of 10 haplotypes were identified from all *Ae. aegypti* populations, labeled as H07, H12, H05, H13, H14, H06, H04, H29, H09, and H30 (GenBank number: ON637920—ON637929). Among these, H06 and H09 were the dominant haplotypes. Molecular cluster analysis of the haplotype sequences indicated that H07, H12, H05, H13, H14, and H06 had only one to two gene mutations compared to the standard *Ae. aegypti cox I* gene sequence (NC_035159.1:1298-2834). In contrast, haplotypes H04, H29, H09, and H30 exhibited multiple gene mutations (Figure 1a). Analysis of haplotype diversity over various years showed a gradual decrease in diversity in Jinghong city, Ruili city, and Mengla city, while diversity remained relatively stable in Daluo port and Lincang city (Figure 1b). The haplotype network analysis further revealed that these haplotypes have evolved into two main evolutionary lineages, with H06 and H09 consistently present in *Ae. aegypti* populations across nearly all regions and years (Figure 1c). Furthermore, a Maximum Likelihood (ML) tree constructed from all 10 haplotypes detected in the present study, along with typical haplotypes from other regions in China and Southeast Asia, including Myanmar, Laos and Thailand, revealed that among the present 10 haplotypes, only H05 exhibits a close phylogenetic relationship with the *Ae. aegypti* strains (OR144886/95/92) originating from Hainan Island and the Leizhou Peninsula in China. The remaining nine haplotypes all demonstrate a relatively close affinity to those from Southeast Asia. Specifically, haplotype H04 is closely related to the *Ae. aegypti* strain from Myanmar (OK510383), haplotype H09 displays a close phylogenetic connection to the *Ae. aegypti* strain from Laos (MW664687), and the other haplotypes are all closely related to the *Ae. aegypti* strains from Thailand (Supplemental Material: Figure S2).

### 3.3. Genetic Structure Within the Three Major Regions of Yunnan Province

**Figure 2:**
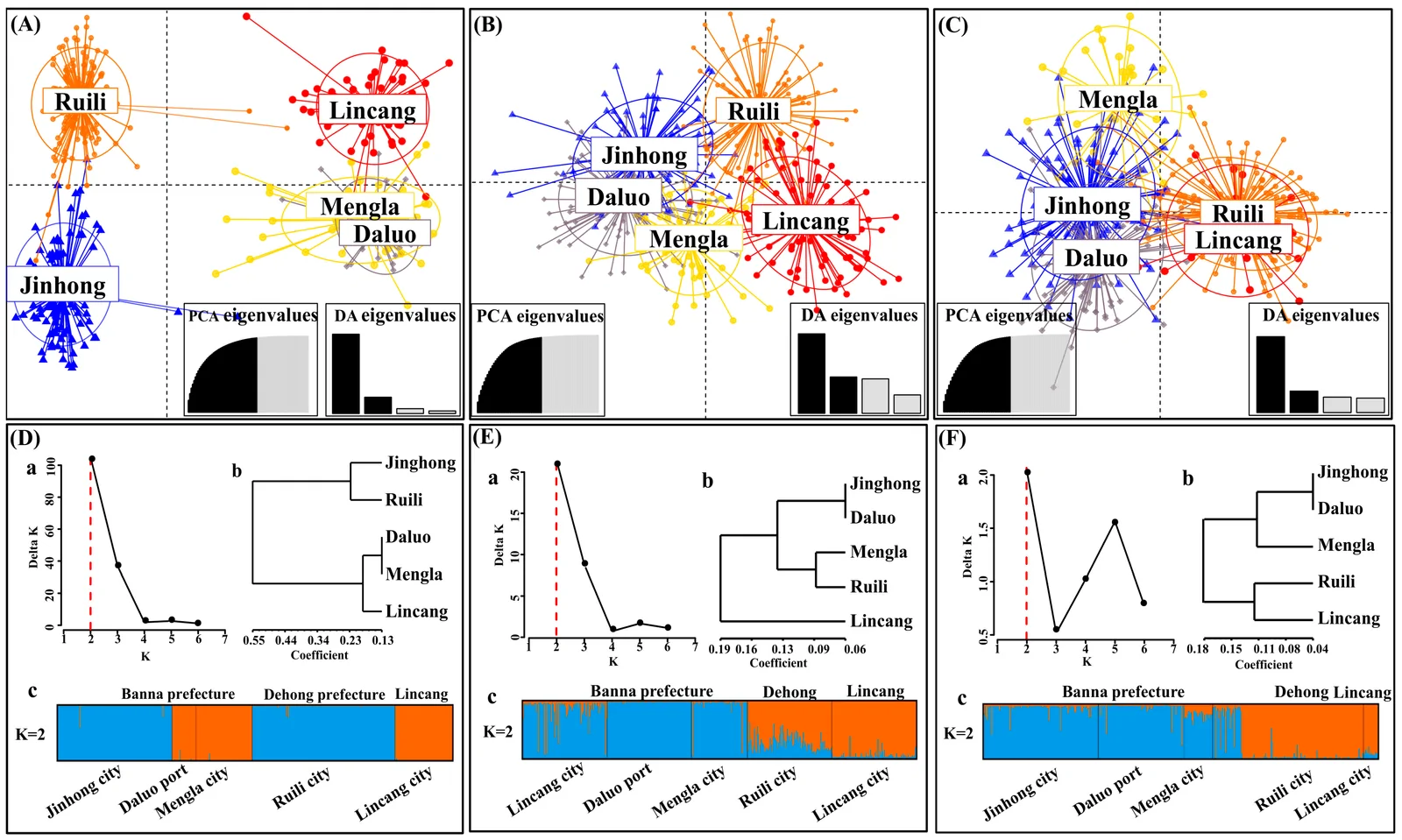
Population structure analysis of *Ae. aegypti* from 2016 to 2018. (**A**–**C**) DAPC analysis of all *Ae. aegypti* populations within five regions from 2016 to 2018; (**D**–**F**) UPGMA clustering and structure analysis of all *Ae. aegypti* populations sampled from 2016 to 2018, where (**a**) displays the optimal K value of *Ae. aegypti* populations; (**b**) displays the UGPMA analysis result of *Ae. aegypti* populations; (**c**) displays the structure analysis result of *Ae. aegypti* populations.

Analysis of population structure revealed notable shifts in the *Ae. aegypti* populations across five sampling sites from 2016 to 2018. In 2016, the optimal number of clusters was determined to be K = 2 using Evanno’s *Δ*K method (Figure 2D(a)). The UPGMA tree and DAPC analysis corroborated this, indicating that the populations belonged to two genetic clusters: one comprising samples from Jinghong city and Ruili city, and the other including samples from Lincang city, Daluo port, and Mengla city (Figure 2A,D(b,c)). In 2017, K = 2 remained the optimal value (Figure 2E(a)), but the cluster composition shifted, with Jinghong city, Daluo port, Mengla city, and Ruili city forming one cluster, and Lincang city forming another (Figure 2B,E(b,c)). By 2018, the populations were again divided into two branches; however, this time, Lincang city and Ruili city clustered together, while Mengla city, Daluo port, and Jinhong city formed the other branch (Figure 2C,F).

### 3.4. Analysis of Molecular Variance (AMOVA) Test of All Ae. aegypti Populations

AMOVA for Yunnan *Ae. aegypti* populations revealed that the largest proportion of molecular variation across 2016–2018 resided among individuals within populations and within individuals, collectively explaining more than 80% of total genetic variance each year (Table 3). The percentage of variance attributed to within-individual variation (*Vd*) was 76.43% in 2016, 65.15% in 2017, and rose to 80.36% in 2018. A striking temporal shift was observed for variation among individuals within populations (*Vc*): this component accounted for only 3.73% of total variance in 2016, but increased substantially to 25.23% in 2017 and remained elevated at 11.26% in 2018. This upward trend indicates increased intra-population genetic variability after 2016. Variation partitioned among regions (*Va*) contributed 11.26% of total variance in 2016, declining to 5.51% (2017) and 5.02% (2018). Variation among populations within regions (*Vb*) represented 8.58% in 2016, followed by 4.11% in 2017 and 3.36% in 2018 (Table 3).

### 3.5. Population Dispersion and Population Structure Similarity Analysis of Ae. aegypti Populations

**Figure 3:**
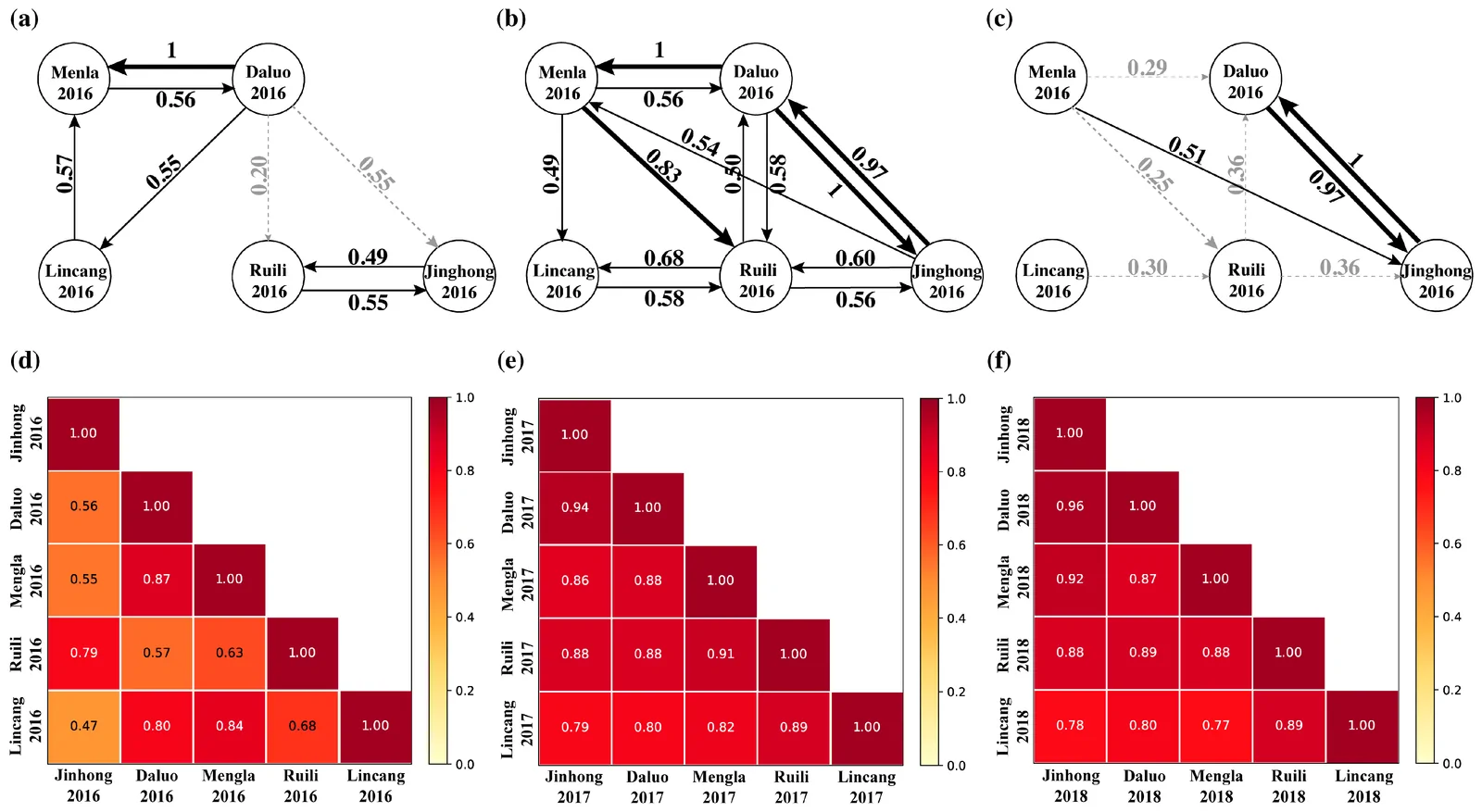
Population similarity and dispersion analysis of *Ae. aegypti* populations among different regions. (**a**–**c**) Population dispersion analysis, where the number is the gene flow value between two regions; (**d**–**f**) population similarity analysis, where the color represents the genetic similarity index (Nei’s genetic identity, *I*) between the populations of two regions. The index *I* ranges from 0 (no shared alleles) to 1 (identical allele frequencies); value closer to 1 indicates greater genetic similarity between populations.

In 2016, two major dispersal cycles were identified across different regions: (1) between Jinghong city and Ruili city, with the number of migrants (Nm) at 0.49 and 0.55, respectively, and (2) a circular dispersal from Mengla city to Daluo port to Lincang city, with Nm values of 0.56, 0.55 and 0.57, respectively. Notably, significant dispersion occurred between Mengla city and Daluo port, with Nm values of 0.56 and 1, respectively (Figure 3a). In 2017, *Ae. aegypti* populations exhibited regional dispersion, with all Nm values surpassing 0.5. Strong dispersion trends were observed from Mengla city to Ruili city (Nm = 0.83), from Daluo port to Mengla city (Nm = 1), and between Jinghong city and Daluo port (Nm = 0.97 and 1, respectively) (Figure 3b). By 2018, significant population dispersion was mainly between Daluo port and Jinghong city (Nm = 0.97 and 1, respectively) and from Mengla city to Jinghong city (Nm = 0.51) (Figure 3c). Concurrently, heat map analysis revealed similar dispersion patterns. In 2016, high genetic similarities were noted between the populations of Daluo port and Mengla city, Mengla city and Lincang city, Daluo port and Lincang city, and Ruili city and Jinghong city, with similarity indices (Nei’s genetic identity, *I*) of 0.87, 0.84, 0.80, and 0.79, respectively (Figure 3d). In 2017, high similarities were found between the populations of Jinghong city and Daluo port, and Ruili city and Mengla city, with indices of 0.94 and 0.91, respectively (Figure 3e). Similarly, in 2018, high population similarity was observed between Jinghong city and Daluo port, and Jinghong city and Mengla city, with indices of 0.96 and 0.92, respectively (Figure 3f).

## 4. Discussion

Genetic diversity is an important genetic index for assessing population adaptability and can be estimated through the observed number of alleles, observed heterozygosity, and expected heterozygosity [45]. Numerous studies have demonstrated that unintentional parental selection and inbreeding during reproduction can diminish genetic diversity in populations [46,47,48,49,50,51,52]. In this study, the *Ho* values for *Ae. aegypti* populations in Jinghong city and Ruili city were consistently lower than the *He* values, indicating a significant excess of homozygotes relative to HWE tests (*p* < 0.05, Supplemental Material: Table S5). Moreover, *F_IS_* values ranged from 0.05 to 0.34, and null alleles were detected at certain loci over the years (Supplemental Material: Tables S2–S4). This suggests that the excess homozygosity may be indicative of inbreeding and slight differentiation among populations. Additionally, the analysis of effective alleles showed that their number initially increased before declining in both populations. Concurrently, haplotype analysis revealed a year-on-year decrease in haplotype diversity. In Mengla city, Daluo port, and Lincang city, population heterozygosity over the monitoring years shifted from *Ho* > *He* to *Ho* < *He*, with *F_IS_* changing from negative to positive. These findings suggest that the *Ae. aegypti* population in Yunnan is undergoing ongoing inbreeding, which is consequently reducing the genetic diversity of the entire population.

Environmental factors, such as temperature and precipitation, significantly influence the distribution and abundance of mosquito populations [53]. Temperature affects mosquito flight activity, host-seeking behavior, and the development of immature stages, while precipitation determines the availability of breeding sites [54,55]. These factors, in turn, impact the population diversity and dispersal of *Aedes* mosquitoes. Sampling strategies may also influence genetic changes in populations over time. As a human-aided dispersal mosquito, *Aedes* typically travel an average distance of 105.69 m on their own, with their geographic spread facilitated by human transportation networks along rivers and highways [56,57,58]. Consequently, the distance and number of sampling sites from rivers or highways may affect the genetic diversity of mosquito populations. Additionally, the frequency of null alleles and the occurrence of Hardy–Weinberg equilibrium within populations can also influence the genetic variation in Aedes mosquitoes over years.

Local mosquito control activities, alongside variations in environmental factors and sampling strategies, may drive genetic changes in mosquito populations [59]. In this study, genetic changes were noted among *Ae. aegypti* populations over different years. In 2017, these populations exhibited widespread dispersion across all sample sites, whereas in 2016 and 2018, significant dispersion was limited to certain sites. This pattern may be linked to mosquito control measures implemented locally from late 2016 to early 2017. Since the first dengue fever outbreak in Ruili city, Yunnan province, in 2015, the disease has continued to cause local outbreaks [60,61,62]. In response, the government has conducted comprehensive investigations into local dengue vectors. These efforts include analyzing the population structure of *Aedes* mosquitoes, examining the sequence characteristics of the dengue virus, assessing mosquito susceptibility to insecticides, and evaluating the effectiveness of governmental mosquito control interventions [7,63,64,65,66]. Such measures might explain the reduced mosquito dispersal across different sampling sites observed in 2018. Meanwhile, stochastic factors could also account for part of the year-to-year variation [67]. Random genetic drift, the intensity of which is inversely related to the effective population size, can generate substantial non-directional fluctuations in allele frequencies and diversity estimates over short time scales, particularly in populations that experience recurrent seasonal bottlenecks and rapid expansions. The domestic *Ae. aegypti* populations in Yunnan Province are characterized by pronounced seasonal fluctuations in abundance and by small, temporary, and unevenly distributed breeding sites, conditions that may amplify the influence of drift on microsatellite-based diversity indices [68]. Moreover, diversity estimates are sensitive to sampling stochasticity, and the limited numbers of individuals and loci typed per site in each year can inflate year-to-year differences unrelated to genuine demographic or evolutionary change [69]. Therefore, in future work, there is also a pressing demand for longer time series and broader spatial sampling to distinguish stochastic variation from deterministic evolutionary processes.

Understanding haplotype structure is crucial for studying species evolution and population dispersion, as rare haplotypes often indicate a spreading population [70,71,72]. In this study, haplotype H06 was the only one consistently detected across all populations over the years, while H09 was absent in the Lincang city population. Haplotypes H14, H29, and H30 appeared solely in Ruili city during 2016 and 2017. Meanwhile, the results of the clustering analysis on haplotypes reveal that only H05 displays a close phylogenetic relationship with the *Ae. aegypti* strains (OR144886/95/92) originating from Hainan Island and the Leizhou Peninsula of China, and the other nine haplotypes all exhibit a relatively close affinity to those from Southeast Asia. This occurrence suggests these haplotypes might have originated from newly invaded *Ae. aegypti* mosquitoes, or could be attributed to the limited sampling size or their inherent rarity. In future monitoring, these rare haplotypes may be valuable indicators for tracking the invasion and dispersion of *Ae. aegypti*.

## 5. Conclusions

This study systematically evaluated the genetic dynamics and changes in the dispersion patterns of *Ae. aegypti* populations in Yunnan province from 2016 to 2018. Given the evolving invasion routes and dispersion patterns of *Ae. aegypti* over years, continuous monitoring remains essential. Additionally, attention should be focused on the intra-provincial spread of mosquitoes, especially between port and urban areas.

## Figures and Tables

**Table 1:** The sample information and characteristics of each *Ae. aegypti* sample site among different regions of Yunnan province, China.

Regions	Sample Site	Geographical Coordinates	Sample Size		
			Year 2016	Year 2017	Year 2018
Banna prefecture	Jinghong city	100°47′58″ E, 22°00′43″ N	120	90	120
	Mengla city	101°41′08″ E, 21°11′35″ N	59	61	60
	Daluo port	100°01′58″ E, 21°40′46″ N	50	91	90
Dehong prefecture	Ruili city	97°51′20″ E, 24°01′04″ N	150	90	163
Lincang area	Lincang city	99°24′10″ E, 23°32′20″ N	60	92	50

**Table 2:** Genetic diversity indices of *Ae. aegypti* populations sampled from five major regions of Yunnan province during 2016 to 2018 based on 9 microsatellite loci.

Sample Site	2016				2017				2018			
	*ne* ^1^	*Ho* ^2^	*He* ^2^	*F_IS_* ^3^	*ne* ^1^	*Ho* ^2^	*He* ^2^	*F_IS_* ^3^	*ne* ^1^	*Ho* ^2^	*He* ^2^	*F_IS_* ^3^
Jinghong	3.30	0.62	0.65	0.05	3.33	0.40	0.61	0.34	2.82	0.56	0.59	0.07
Dehong	3.18	0.55	0.64	0.14	3.26	0.41	0.62	0.27	2.87	0.45	0.57	0.18
Mengla	2.96	0.65	0.61	−0.07	2.65	0.43	0.55	0.23	2.24	0.47	0.53	0.12
Daluo	2.93	0.62	0.59	−0.04	2.82	0.43	0.57	0.25	2.75	0.53	0.58	0.09
Lincang	2.78	0.61	0.58	−0.06	3.33	0.44	0.61	0.29	2.16	0.41	0.50	0.23

^1^ *ne*: effective number of alleles [42]. ^2^ *Ho* and *He*: *Ho*, observed heterozygosity; *He*, expected heterozygosity [43]. ^3^ *F_IS_*: inbreeding coefficient [44].

**Table 3:** Analysis of molecular variance (AMOVA) test of all *Ae. aegypti* populations sampled from Yunnan province from 2016 to 2018.

Source of Variation	2016		2017		2018	
	Variance Components	Percentage of Variation	Variance Components	Percentage of Variation	Variance Components	Percentage of Variation
Among regions	0.397Va	11.26	0.161Va	5.51	0.120Va	5.02
Among populations within regions	0.302Vb	8.58	0.120Vb	4.11	0.080Vb	3.36
Among individuals Within populations	0.132Vc	3.73	0.739Vc	25.23	0.270Vc	11.26
Within individuals	2.693Vd	76.43	1.908Vd	65.15	1.924Vd	80.36

1. Significance tests (10,100 permutations); 2. For the year 2016: F_CT_(*Va*) = 0.113, F_SC_(*Vb*) = 0.097 ***, *F_IS_*(*Vc*) = 0.047 ***, F_IT_(*Vd*) = 0.236 ***. For the year 2017: F_CT_(*Va*) = 0.055, F_SC_(*Vb*) = 0.043 ***, *F_IS_*(*Vc*) = 0.279 ***, F_IT_(*Vd*) = 0.348 ***. For the year 2018: F_CT_(*Va*) = 0.050, F_SC_(*Vb*) = 0.035 ***, *F_IS_*(*Vc*) = 0.123 ***, F_IT_(*Vd*) = 0.196 ***. All F-statistic values marked with *** are statistically significant at *p* < 0.0001.

## Data Availability

The datasets supporting the conclusions of this article are included within the article. Given that this study was carried out within the framework of a single, continuously funded project in collaboration with Shi et al. (2017) [23], we extracted partial mitochondrial gene *cox I* and microsatellite data originally reported previously for inclusion in the present research.

## Supplementary Materials

The following supporting information can be downloaded at: https://www.mdpi.com/article/10.3390/insects17101087/s1, Table S1: A detailed description of the sampling biotopes including location, main vegetation, soil type, etc., and water bodies; Table S2: Genetic diversity and null alleles test of 9 microsatellite loci among *Ae. aegypti* populations sampled from Yunnan province at 2016; Table S3: Genetic diversity and null alleles test of 9 microsatellite loci among *Ae. aegypti* populations sampled from Yunnan province at 2017; Table S4: Genetic diversity and null alleles test of 9 microsatellite loci among *Ae. aegypti* populations sampled from Yunnan province at 2018; Table S5: Hardy–Weinberg equilibrium test among *Ae. aegypti* populations sampled from Yunnan province during 2016 to 2018; Figure S1: A Sampling map depicting Aedes aegypti populations in Yunnan Province from 2016 to 2018; Figure S2: The Maximum Likelihood (ML) Tree of all 10 haplotypes detected in present study with the typical haplotypes from other sites of China and Southeast Asia including Myanmar, Laos and Thailand; Data S1: Original microsatellite and *cox I* data for 2016 *Ae. aegypti* populations.

## Abbreviations

The following abbreviations are used in this manuscript:

*Ae.*	*Aedes*
*An.*	*Anopheles*
CHIKV	Chikungunya virus
DENV	Dengue virus
ZIKV	Zika virus
*c**ox I*	Mitochondrial cytochrome c oxidase subunit 1
*Ho*	Observed heterozygosity
*He*	Expected heterozygosity
*I*	Nei’s genetic identity
HWE test	Hardy–Weinberg equilibrium test
AMOVA test	Analysis of molecular variation test
*ne*	Effective number of alleles
*F_IS_*(*Vc*)	Inbreeding coefficient
F_ST_	Fixation Index
F_CT_(*Va*)	Genetic differences among regions
F_SC_(*Vb*)	Genetic differences among populations within regions
F_IT_(*Vd*)	Genetic differences within individuals
PCA	Principal component analysis
DAPC	Discriminant analysis of principal components
Nm	Number of migrants
UGPMA	Unweighted Pair Group Method with Arithmetic Mean
ML	Maximum Likelihood
