# Supplementary material for: Genetic Dynamics and Dispersion Pattern Changes in Aedes aegypti Populations (Diptera: Culicidae) in Yunnan Province, China, over Three Consecutive Years
Source: Insects. 2026 Oct 20;17(10):1087. doi: 10.3390/insects17101087 (PMC13705575; doi:10.3390/insects17101087)
Supplement: Supplementary file 1 [file insects-17-01087-s001.zip › SUPPLE~1.pdf]

Supplemental material: Table S1. A detailed description of the sampling biotopes including location, main vegetation, soil type, etc. and water bodies.

Sampling site	Location	Main vegetation	Soil type	water bodies
Jinghong city	Core of Banna prefecture; Adjacent to the Mekong (Lancang) River	Tropical seasonal rainforest remnants, rubber plantations	Lateritic red soil (latosolic)	Urban domestic water storage tanks; Discarded tires; ceramic jars and flower pots
Mengla city	Eastern border of Banna prefecture; Bordering Laos	Tropical rainforest, rubber and tea plantations	Lateritic soil	Village water jars; Natural tree holes; Discarded tires
Daluo port	Border town of Banna prefecture; Bordering Myanmar	Subtropical evergreen broadleaf forest, rubber plantations	Lateritic red soil	Discarded plastic containers; Discarded tires and Village water containers
Ruili city	Western city of Dehong prefecture; Bordering Myanmar	Subtropical monsoon forest, bamboo groves	Red soil & lateritic red soil	Urban water tanks; Discarded tires; flower pots; Stagnant water on the tarp
Lincang city	Southwestern Yunnan; Relatively inland; Surrounded by high mountains;	Subtropical evergreen broadleaf forest, tea plantations	Mountain red-yellow soil	Village water jars; Stagnant water on the tarp; Discarded plastic containers

Supplemental material: Table S2. Genetic diversity and null alleles test of 9 microsatellite loci among *Ae. aegypti* populations sampled from Yunnan province at 2016.

No.	Locus name	Size	Allele number	Gene Diversity	PIC	Null Allele
1	Aa1	960	9	0.988	0.427	-
2	Aa3	960	15	2.116	0.823	-
3	Aa5	960	20	1.550	0.621	yes
4	Aa12	960	21	0.922	0.329	yes
5	Aa16	960	8	0.980	0.462	-
6	Aa23	956	22	2.249	0.846	-
7	Aa30	960	14	1.634	0.727	-
8	Aa44	956	10	1.509	0.665	-
9	Aa45	958	11	1.190	0.561	-
	Average	959	14	1.460	0.607	

Supplemental material: Table S3. Genetic diversity and null alleles test of 9 microsatellite loci among *Ae. aegypti* populations sampled from Yunnan province at 2017.

No.	Locus name	Size	Allele number	Gene Diversity	PIC	Null Allele
1	Aa1	960	7	0.644	0.392	-
2	Aa3	960	16	1.421	0.804	-
3	Aa5	960	13	1.119	0.629	-
4	Aa12	960	12	0.685	0.633	-
5	Aa16	960	9	0.998	0.679	-
6	Aa23	956	20	1.597	0.886	-
7	Aa30	960	10	1.193	0.657	yes
8	Aa44	956	11	1.187	0.614	yes
9	Aa45	958	16	1.380	0.755	-
	Average	959	12	1.136	0.672	

Supplemental material: Table S4. Genetic diversity and null alleles test of 9 microsatellite loci among *Ae. aegypti* populations sampled from Yunnan province at 2018.

No.	Locus name	Size	Allele number	Gene Diversity	PIC	Null Allele
1	Aa1	884	8	0.881	0.389	-
2	Aa3	886	11	1.848	0.778	yes
3	Aa5	876	6	1.025	0.494	-
4	Aa12	886	14	0.469	0.180	-
5	Aa16	886	5	0.914	0.441	-
6	Aa23	844	16	1.845	0.747	-
7	Aa30	886	13	1.582	0.719	-
8	Aa44	886	9	1.583	0.717	-
9	Aa45	886	8	1.220	0.591	-
	Average	880	10	1.263	0.562	

Supplemental material: Table S5. Hardy-Weinberg equilibrium test among *Ae. aegypti* populations sampled from Yunnan province during 2016 to 2018.

Sample site	HWE	df	Chi-square	Prob
Jinghong City2016	0.062	9	197.76	0.0000
Menla City2016	-0.100	9	133.47	0.0000
Daluo Port2016	-0.196	9	61.27	0.0000
Ruili City2016	0.214	9	223.20	0.0000
Lincang City2016	-0.102	9	35.35	0.0001
Jinghong City2017	0.387	9	84.59	0.0000
Menla City2017	0.278	9	110.46	0.0000
Daluo Port2017	0.322	9	71.13	0.0000
Ruili City2017	0.339	9	138.86	0.0000
Lincang City2017	0.362	9	100.45	0.0000
Jinghong City2018	0.022	9	49.40	0.0000
Menla City2018	0.120	9	23.44	0.0050
Daluo Port2018	0.086	9	40.51	0.0000
Ruili City2018	0.239	9	55.45	0.0000
Lincang City2018	0.117	9	10.82	0.2883

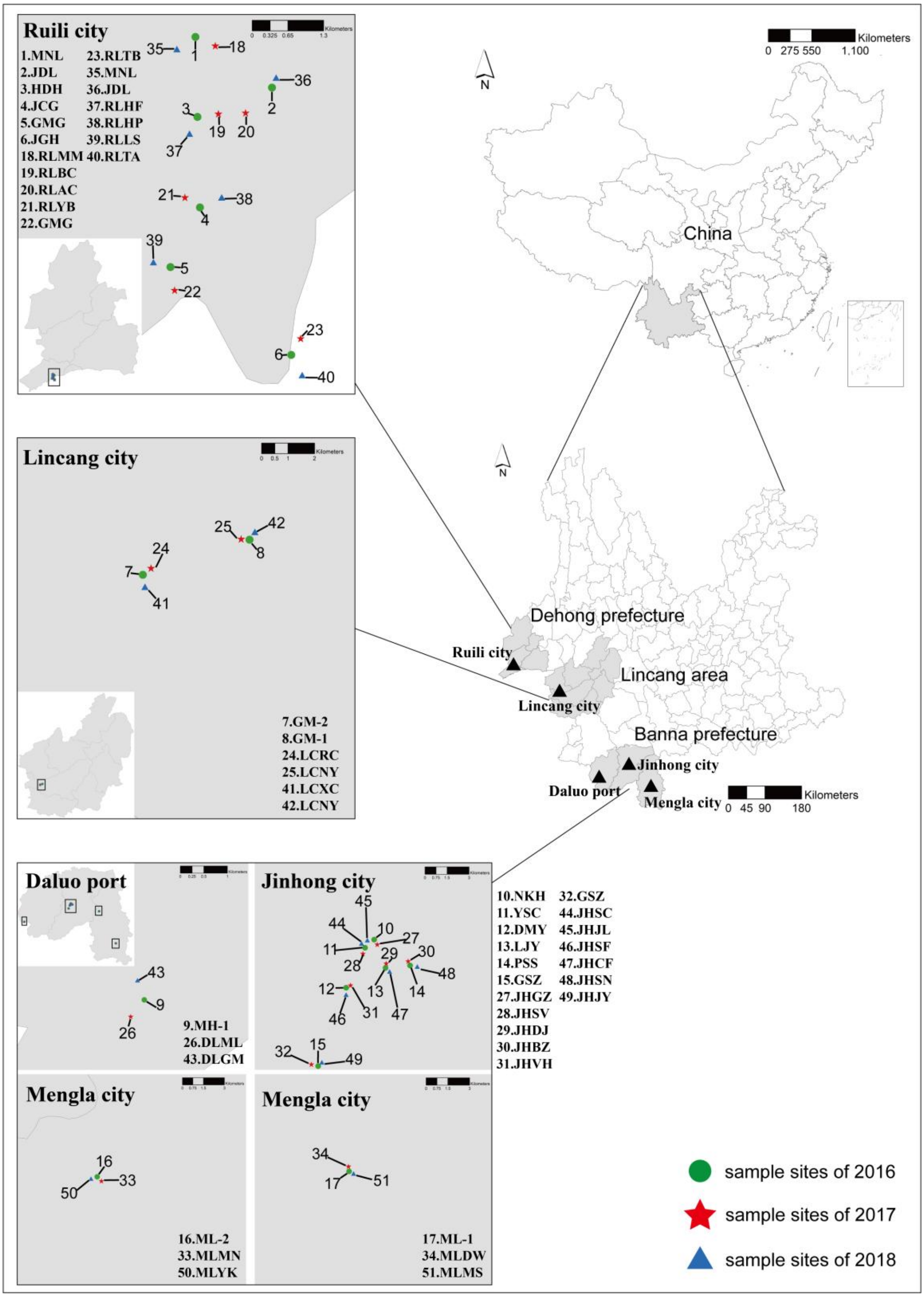

Supplemental material: Figure S1. A Sampling map depicting *Aedes aegypti* populations in Yunnan Province from 2016 to 2018. The specific sampling sites denoted by the codes in figure are as follows: Jinhong city 2016 contains Linjiangyuan (LJY), Nonken Hospital (NKH), Gasa town (GSZ), Damanyao (DMY), Yunshu Company (YSC), and Pi'er Park (PSS); Ruili city 2016 contains Munao Road (MNL), Jindian Road (JDL), Jiegao Freight Yard (JGH), Guomen bridge (GMG), Jincheng Steel Plant (JCG), and Hongda Freight Yard (HDH); Mengla city 2016 contains Nanla Middle School (ML-1) and Botanical

Garden (ML-2); Daluo port 2016 contains Daluo (MH-1); Lincang city 2016 contains Mengding-1 (GM-1) and Mengding-2 (GM-2); Jinhong city 2017 contains Daijiangnan(JHDJ), Gaozhuang (JHGZ), Gasa town (GSZ), VOCO hotel (JHVH), Suhu village (JHSV) and Banzhang village (JHBZ); Ruili city 2017 contains Mengmao Village (RLMM), Ancient City (RLAC), Television Building (RLTB), Guomen bridge (GMG), Yingbinlou Hotel (RLYB) and Biancheng Street(RLBC); Mengla city 2017 contains Daiweixiu (MLDW) and Mini parking site (MLMN); Daluo port 2017 contains Meilongka (DLML); Lincang city 2017 contains Nongyang Village (LCNY) and Rongchang Repair Shop(LCRC); Jinhong city 2018 contains Cement Factory (JHCF), Jinglan Area (JHJL), Jingyuan Area (JHJY), Soap Factory (JHSF), Sports Center (JHSC) and Starlight Night Market (JHSN); Ruili city 2018 contains Munao Road (MNL), Jindian Road (JDL), Tang'ai Jewelry Store (RLTA), Linsen Hotel (RLLS), Hepai Village (RLHP) and Hefei Village (RLHF); Mengla city 2018 contains No.2 Middle School (MLMS) and Youlan Hotel (MLYL); Daluo port 2018 contains Daluo Border Gate(DLGM); Lincang city 2018 contains Nongyang Village (LCNY) and Xiacheng Nongyang (LCXC).

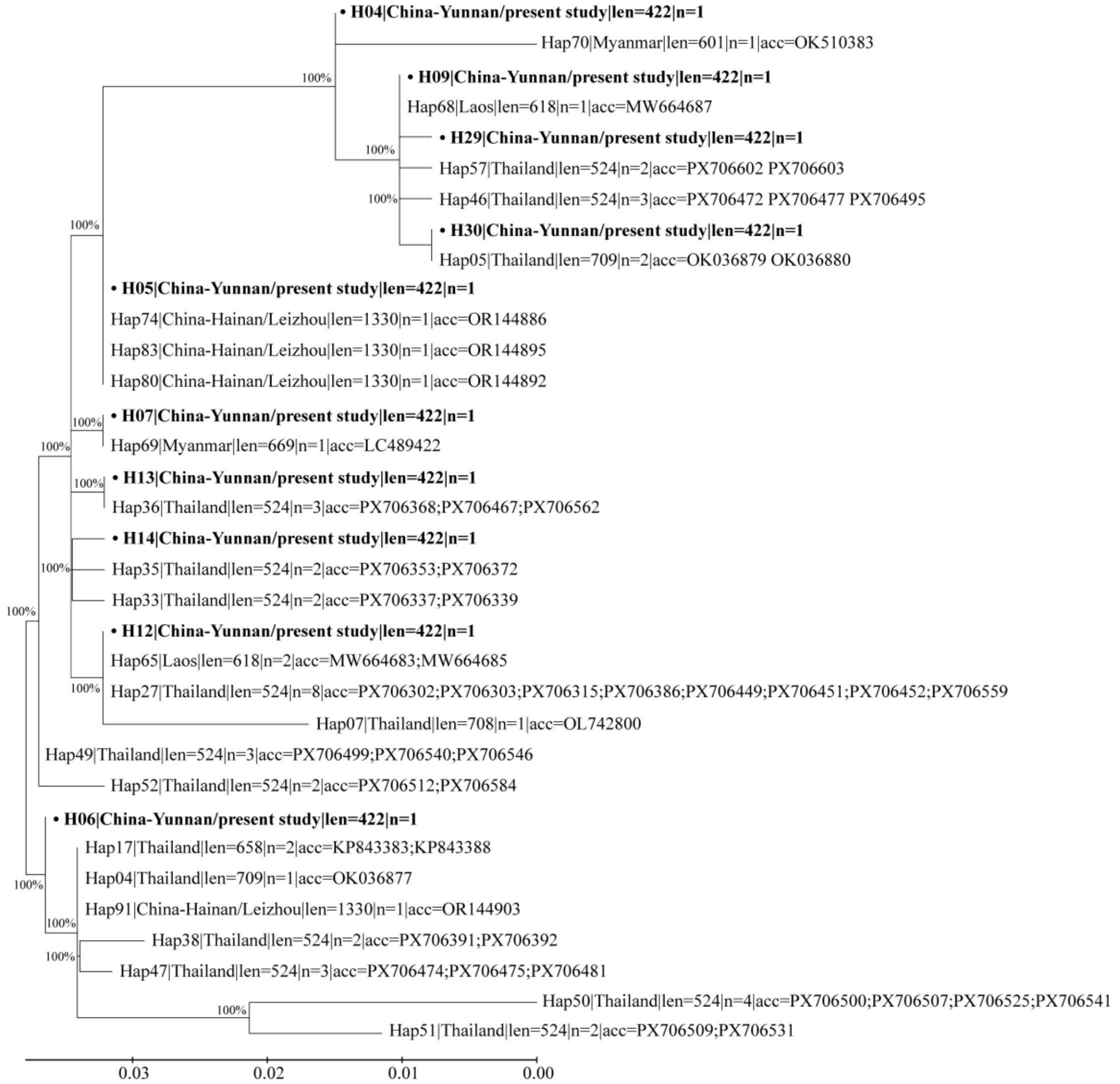

Supplemental material: Figure S2. The Maximum Likelihood (ML) Tree of all 10 haplotypes detected in present study with the typical haplotypes from other sites of China and Southeast Asia including Myanmar, Laos and Thailand.
